# Association of FSHR and DENND1A polymorphisms with polycystic ovary syndrome: a meta-analysis

**DOI:** 10.5935/1518-0557.20220043

**Published:** 2023

**Authors:** Celina Bakke Larsen, Erik Kudela, Kamil Biringer

**Affiliations:** 1 Department of Gynecology and Obstetrics, Jessenius Faculty of Medicine in Martin, Comenius University in Bratislava, Slovakia

**Keywords:** polycystic ovary syndrome, follicle stimulating hormone gene, DENND1A gene, genetics, meta-analysis

## Abstract

**Objective:**

Multiple genetic variants have been studied for years to try to find an association with polycystic ovary syndrome (PCOS). This meta-analysis will investigate if there are associations between increased risk of PCOS and rs6165 polymorphism in follicle stimulating hormone receptor (FSHR) gene and rs2479106 polymorphism in differentially expressed in Differentially Expressed in Normal and Neoplastic Development Isoform 1A (DENND1A) gene.

**Methods:**

Studies were identified from PubMed library, and case-control studies with correct polymorphisms and available genotype frequencies were included. The statistical analysis is done in Review Manager 5.3, and odds ratio (OR) with corresponding 95% confidence interval (CI) was calculated to see if any association with PCOS exists.

**Results:**

In the study of FSHR gene, eight articles with 1539 cases and 1877 controls were included. No relations were found between PCOS and rs6165 polymorphism in neither the allelic (OR=1.07, 95% CI=0.97-1.19, *p*=0.18), recessive (OR=1.21, 95% CI=0.98-1.50, *p*=0.07) nor the dominant (OR=1.05, 95% CI=0.91-1.20, *p*=0.53) model. The rs2479106 polymorphism in DENND1A gene included 10 studies with 3627 cases and 20325 controls. Only the Asian subgroup in the recessive model (OR=1.84, 95% CI=1.19-2.85, *p*=0.006) showed a positive relation with PCOS, while associations were not found within the overall results in the allelic (OR=1.09, 95% CI=0.98-1.21, *p*=0.10), recessive (OR=1.26, 95% CI=0.73-2.19, *p*=0.41) or the dominant (OR=1.31, 95% CI=1.00-1.71, *p*=0.05) model.

**Conclusions:**

This meta-analysis suggests that rs2479106 polymorphism in DENND1A gene is associated with increased risk of PCOS in the Asian population. No relations were found with increased risk of PCOS and rs6165 polymorphism in FSHR gene.

## INTRODUCTION

Polycystic ovary syndrome (PCOS) is one of the most common endocrine disorders in women of reproductive age with 12 to 18% affected worldwide (Joham *et al.*, 2016). The clinical presentation is heterogenous, and different criteria and phenotypes are used depending on the presence or absence of features like hyperandrogenism, ovulatory dysfunction and polycystic ovarian morphology (Azziz *et al*., 2016). It is a disorder with a poor understood etiology and its phenotype varies by race and ethnicity, and is exacerbated by obesity (Dumesic *et al.*, 2015). Women with PCOS present a higher risk of dyslipidemia, type 2 diabetes mellitus, cardiovascular complications, excessive weight and metabolic syndrome compared to other women (Azziz *et al*., 2016; Carvalho *et al.*, 2018). PCOS is the most frequent cause of hyperandrogenism and oligo-anovulation in females in reproductive age and both of these features can cause social and psychological problems. As a complication to chronic anovulation, infertility can also be a consequence (Norman *et al*., 2007).

Genetics have been important in the study of PCOS due to its complexity and because several genes are involved in its etiology. The genetic etiology was first questioned by Cooper *et al.* (1968) and multiple genes have been studied to try to find a connection to PCOS. One gene of interest is follicle-stimulating hormone receptor (FSHR) gene. FSHR is one of the receptors where polymorphisms can be involved in the etiology of PCOS due to its involvement in the development of gonads. It encodes a G-coupled protein receptor which is expressed in granulosa cells, and is located at chromosome 2p21-p16 and consists of 10 exons (Gromoll & Simoni, 2005). FSHR encode follicle-stimulating hormone (FSH), and any dysfunctions in this receptor may disturb the function of follicles and ovaries (Ajmal *et al.*, 2019). Mutations which inactivate FSHR will lead to hypergonadotropic hypogonadism, which can cause the follicles to stagnate in the preantral state (McAllister *et al.*, 2015). In 2005, 731 (single nuclear polymorphisms) SNPs were identified in the FSHR gene (Gromoll & Simoni, 2005) in 2014 this number was 900 SNPs (Simoni & Casarini, 2014) and in 2019, around 1800 SNPs of FSHR gene have been reported. Only eight of these SNPs are located in the coding region, and seven of them occur in exon 10. Six of the SNPs result in an amino acid substitution and one of the most common SNPs are Ala307Thr (rs6165) (Laven, 2019) where threonine (Thr) is replaced by alanine (Ala) (Simoni & Casarini, 2014).

The next gene in this study is Differentially Expressed in Normal and Neoplastic Development Isoform 1A (DENND1A) gene, also called differentially expressed in normal and neoplastic development isoform 1A gene. It has been identified as a risk marker for PCOS and is located at chromosome 9q33.3. This is a poorly characterized protein that regulate Rab-mediated membrane trafficking pathways (Mykhalchenko *et al.*, 2017) and is placed inside the cytoplasm and in the nuclei of ovarian theca cells. Two variants, DENND1A variant 1 (DENND1A.V1) and DENND1A variant 2 (DENND1A.V2), have been found, and the first variant encodes a protein with a proline-rich domain, while the second variant lack this domain, but include a C-terminal 33-aa sequence (Crespo *et al.*, 2018).

McAllister *et al*. (2014) found that DENND1A.V2 is elevated in theca cells in PCOS patients. They tried to force an overexpression of DENND1A.V2 in normal theca cells, which resulted in an increased androgen synthesis compatible with the elevated androgen profile we can find in PCOS women. Contrarily, when they tried to reduce these levels, the androgen synthesis was decreased, and the authors concluded that DENND1A plays a role in hyperandrogenemia in women with PCOS (McAllister *et al.*, 2014). The reason for this increased expression has not been identified, but by its localization, DENND1A.V2 can influence both gonadotropin and insulin receptors, and thereby increase the expression of steroidogenic enzymes. This variant of DENND1A gene has likewise been localized in the reticular zone in adrenal glands, which also can cause an abnormal androgen synthesis in women with PCOS (McAllister *et al.*, 2015).

Many studies have tried to find an association with these genes and PCOS, both in Europe and Asia. Considering that the results have been controversial, I decided to do a meta-analysis with rs6165 and rs2479106 SNPs in FSHR gene and DENND1A gene respectively to see if they have an association to PCOS.

## MATERIAL AND METHODS

### Data sources and selection criteria

Pubmed was used to identify studies, and the relevant search was done in January and February 2020. The keywords correlated with the polymorphisms of interest and the following words were used: “FSHR”, “rs6165”, “DENND1A”, “rs2479106”, “polymorphism”, “gene” all coupled with “Polycystic ovary syndrome” or “PCOS”. The primary outcome was PCOS women with the risk allele belonging to the polymorphisms in this study.

Studies were included in this meta-analysis only if following criteria were met: (1) case-control study where patients are not related to controls; (2) rs6165 or rs2479106 polymorphisms; (3) clear criteria of diagnosis; (4) genotype frequencies of genes were available, both patients and controls; (5) the frequency genotype distribution was consistent with Hardy-Weinberg equilibrium (HWE). Studies with these criteria were excluded: (1) animal studies; (2) foreign languages; (3) meta-analysis, reviews, family studies; (4) not sufficient data; (5) other polymorphisms.

The meta-analysis is registered in the International prospective register of systematic reviews (PROSPERO) under designation CRD42021229235.

### Data extraction

The extracted data from each study included: the name of the first author, year of publication, country and ethnicity of the population in the study, diagnosis criteria of PCOS, sample size of patients and controls, mean age and body mass index (BMI) of patients and controls and distribution of genotype- and allele frequencies.

### Statistical analysis

In this research, Review Manager 5.3 (provided by Cochrane Collaboration) was used to make forest plots for each of the polymorphisms. The association between polymorphism of interest and PCOS was evaluated by odds ratio (OR) with corresponding 95% confidence interval (CI) and *p*-value. *P*-values <0.05 is considered significant. To find the pooled OR calculation, each polymorphism had three genetic models; allelic model (risk allele *vs*. non-risk allele), recessive model (homozygote risk-allele genotype *vs*. heterozygote genotype + homozygote non-risk genotype) and dominant model (homozygote risk-allele genotype + heterozygote genotype *vs*. homozygote non-risk allele genotype).

Heterogeneity is tested with Cochrane´s Q test, which will give a I^2^ value and its corresponding *p*-value. The choice of forest plot model depends on the heterogeneity results, and according to Cochrane handbook, I^2^<50% and *p*≥0.1 show no heterogeneity, and a Mantel-Haenszel fixed-effect model is chosen. I^2^≥50% and *p*<0.1 are indications of heterogeneity and random-effects model are preferred. If a model shows heterogeneity, subgroup analysis based on ethnicity will be investigated to see if this is the reason for variability.

Hardy-Weinberg Equilibrium (HWE) is tested in all controls by comparing the observed genotypes frequencies to the expected ones in χ^2^ Goodness-of-Fit test. *P*-values <0.05 are not consistent with HWE, and are not included in this analysis.

Publication bias is also estimated in this study, and studies that are not statistically significant but have positive results, may bias the result in a meta-analysis and can lead to a false-positive result. In this analysis, a funnel plot made in Review manager is used to estimate publication bias.

## RESULTS

### Characteristics of included studies

**Figure 1:**
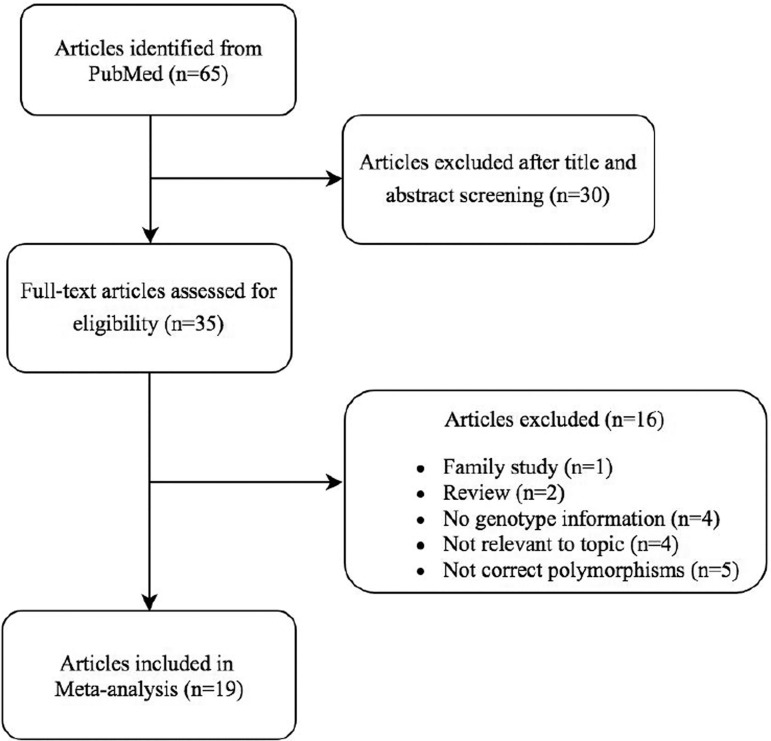
Flowchart of included articles in meta-analysis.

In the identification of articles concerning FSHR and DENND1A polymorphisms, a total of 65 studies from Pubmed were identified. 30 of these were excluded after reading the title and abstract due to irrelevance to the chosen genes or polymorphisms. 35 full-text texts were assessed for eligibility, and 16 articles were excluded because of family study (n=1), review (n=2), no information about genotype (n=4), not relevant to topic (n=4) or not correct polymorphisms (n=5). Finally, 19 articles have been used in this meta-analysis (Flowchart in Figure 1), and all included articles are in English language.

The polymorphism related to FSHR gene is rs6165 (Ala307Thr). A total of 1539 cases of PCOS patients and 1877 controls from eight studies are included, and the articles were published from 2009 to 2018. Extracted information include last name of author, year of publication, country, ethnicity, the definition criteria, sample size, mean age and BMI for both PCOS patients and controls (Table 1). 2/8 studies have Caucasian ethnicity (Unsal *et al.*, 2009; Branavan *et al.*, 2018) while the last six have an Asian origin (Gu *et al.*, 2010; Fu *et al.*, 2013; Singhasena *et al.*, 2014; Wu *et al.*, 2014; Liaqat *et al.*, 2015; Kim *et al.*, 2017). The Rotterdam criteria is used in 7/8 studies, which include two out of three of the PCOS features: oligomenorrhea, hyperandrogenism and polycystic ovaries (Rotterdam ESHRE/ASRM-Sponsored PCOS Consensus Workshop Group, 2004). In the study of Branavan *et al*. (2018), patients with PCOS had to have all the three diagnostic features (oligomenorrhea, hyperandrogenism and polycystic ovaries) to be included.

**Table 1:** Characteristics of studies included the meta-analysis of FSHR and DENND1A gene.

Gene	Study	Country	Ethnicity	Definition criteria	Sample size		Mean age (years)		Mean BMI (kg/m^2^)	
					PCOS	Control	PCOS	Control	PCOS	Control
**FSHR**	Branavan *et al*. (2018)	Sri Lanka	Caucasian	OM, PO, HA	55	110	24.67±0.883	33.80±0.528	26.89±0.716	25.25±0.344
	Kim *et al*. (2017)	South Korea	Asian	Rotterdam	377	388	28.5±4.9	28.5±4.9	22.2±4.0	20.1±2.3
	Liaqat *et al*. (2015)	Pakistan	Asian	Rotterdam	96	96	26.87±4.42	26.02±3.521	31.10±1.47	30.49±1.66
	Wu *et al*. (2014)	China	Asian	Rotterdam	215	205	30.02±4.92	31.06±4.89	24.49±4.26	22.77±3.96
	Singhasena *et al*. (2014)	Thailand	Asian	Rotterdam	133	132	26.6±5.3	30±10	24.3±6.1	NA
	Fu *et al*. (2013)	China	Asian	Rotterdam	384	768	28.3±2.2	27.7±1.8	21.55±1.25	20.45±0.75
	Gu *et al*. (2010)	South Korea	Asian	Rotterdam	235	128	NA	NA	22.96±3.86	20.95±2.49
	Unsal *et al*. (2009)	Turkey	Caucasian	Rotterdam	44	50	14.5±1.3	14.0±3.3	25.0±5.5	20.7±4.2
**DENND1A**	Zhu *et al*. (2020)	China	Asian	Rotterdam	346	225	29.52±3.97	34.38±2.29	29.69±3.14	22.03±2.35
	Xia *et al*. (2019)	China	Asian	Rotterdam	163	171	28.19±3.57	28.54±3.31	22.67±2.21	22.16±2.62
	Dallel *et al*. (2018)	Tunisia	Arab	Rotterdam	320	446	30.85±4.7	31.83±6.0	29.22±6.1	25.93±5.4
	Gammoh *et al*. (2015)	Bahrain	Asian	Rotterdam	191	202	28.5±5.8	26.6±6.7	29.9±6.1	26.0±5.5
	Xu *et al*. (2015)	China	Asian	Rotterdam	800	1110	26.5±3.6	26.8±3.8	23.5±4.3	20.4±2.2
	Eriksen *et al*. (2012)	Denmark	Caucasian	Rotterdam	168	248	29 (24-33)	25 (23-27)	26.2 (22.8-30.8)	23.3 (21.6-24.8)
	Welt *et al*. (2012)	Iceland US (Boston) US (Chicago)	Caucasian Caucasian	NIH NIH NIH	376 559 201	16947 477 188	18-45 18-45 18-45	NA 18-45 >18	NA NA NA	NA NA NA
			Caucasian							
	Lerchbaum *et al*. (2011)	Austria	Caucasian	Rotterdam	503	311	27 (23-31)	29 (26-36)	24.2 (21.2-29.0)	24.4 (20.9-29.2)
	Lerchbaum *et al*. (2011)	Austria	Caucasian	Rotterdam	503	311	27 (23-31)	29 (26-36)	24.2 (21.2-29.0)	24.4 (20.9-29.2)

(FSHR - follicle stimulating hormone receptor, DENND1A - Differentially Expressed in Normal and Neoplastic Development isoform 1A, BMI - body mass index, PCOS - polycystic ovary syndrome, OM - oligomenorrhea, PO - polycystic ovaries, HA - hyperandrogenism, NA - not available, NIH - National Institutes of Health, US - United States)

In the meta-analysis of polymorphism rs2479106 in DENND1A gene, 3627 PCOS patients and 20325 controls are included in eight articles with a total of 10 studies. The higher number of studies is because the article by Welt *et al*. (2012) include populations from Boston, Chicago and Iceland, but they are separately analyzed in this paper. The articles were published from 2011 to 2020, and extracted information is first name of author, year of publication, country and ethnicity of PCOS patients and controls, definition criteria, sample size, mean age and mean BMI for both PCOS patients and controls (Table 1). Four of the articles have an Asian population (Gammoh *et al.*, 2015; Xu *et al.*, 2015; Xia *et al.*, 2019; Zhu *et al.*, 2020) one article has an Arab population (Dallel *et al.,* 2018) while the last three articles have included women with a Caucasian ethnicity (Lerchbaum *et al.,* 2011; Eriksen *et al.*, 2012; Welt *et al.*, 2012). Seven of the studies used the Rotterdam criteria, while one article by Welt *et al*. (2012) used the NIH criteria which includes the presence of chronic anovulation and hyperandrogenism (Azziz, 2005).

### Meta-analysis

**Figure 2:**
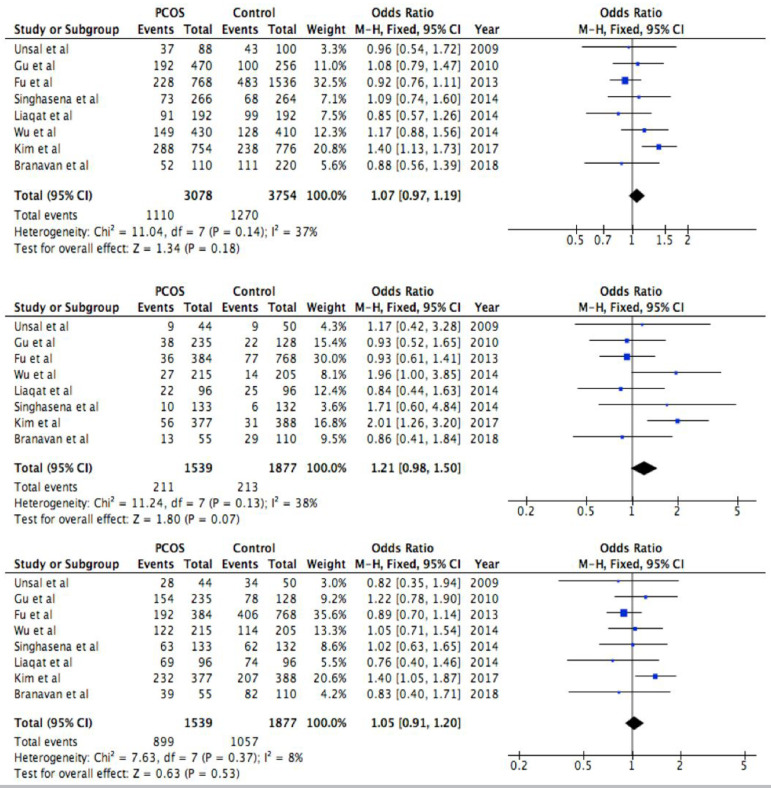
Forest plot of the association between rs6165 polymorphism and PCOS in allelic model (first forest plot, Ala *vs*. Thr), recessive model (second forest plot, Ala/Ala *vs*. Ala/Thr+Thr/Thr) and dominant model (third forest plot, Ala/Ala+Ala/Thr *vs*. Thr/Thr). Solid squares are OR from individual studies, horizontal lines are 95% CI. Pooled OR with 95% CI are presented in diamonds.

The association between rs6165 polymorphism in FSHR gene and the risk of PCOS has been analyzed based on allele and genotype frequencies with HWE values (Table 2), and pooled OR with 95% CI and heterogeneity values has been investigated. No relations were discovered between rs6165 polymorphism and increased risk of PCOS in any of the models, seen in forest plots in Figure 2 (allelic model: OR=1.07, 95% CI=0.97-1.19, *p*=0.18; recessive model: OR=1.21, 95% CI=0.98-1.50, *p*=0.07; dominant model: OR=1.05, 95% CI: 0.91-1.20, *p*=0.53). A fixed model was used in each of them because the heterogeneity test did not show significant heterogeneity (allelic model: I^2^=37%, *p*=0.14; recessive model: I^2^=38%, *p*=0.13; dominant model: I^2^=8%, *p*=0.37). Because of these values, subgroup analysis was not necessary.

**Table 2:** Allele and genotype frequencies with HWE values of rs6165 polymorphism in FSHR gene.

Study	PCOS		Control		PCOS			Control			HWE
	Thr	Ala	Thr	Ala	Thr/Thr	Thr/Ala	Ala/Ala	Thr/Thr	Thr/Ala	Ala/Ala	
Branavan *et al*. (2018)	58	52	109	111	16	26	13	28	53	29	0.70
Kim *et al*. (2017)	466	288	538	238	145	176	56	181	176	31	0.19
Wu *et al*. (2014)	281	149	282	128	93	95	27	91	100	14	0.05
Liaqat *et al*. (2015)	101	91	93	99	27	47	22	22	49	25	0.83
Singhasena *et al*. (2014)	193	73	196	68	70	53	10	70	56	6	0.21
Fu *et al*. (2013)	540	228	1053	483	192	156	36	362	329	77	0.86
Gu *et al*. (2010)	278	192	156	100	81	116	38	50	56	22	0.36
Unsal *et a*l. (2009)	51	37	57	43	16	19	9	16	25	9	0.89

(HWE - Hardy-Weinberg equilibrium, FSHR - follicle stimulating hormone receptor, PCOS - polycystic ovary syndrome, Thr - threonine - Ala - alanine).

**Figure 3:**
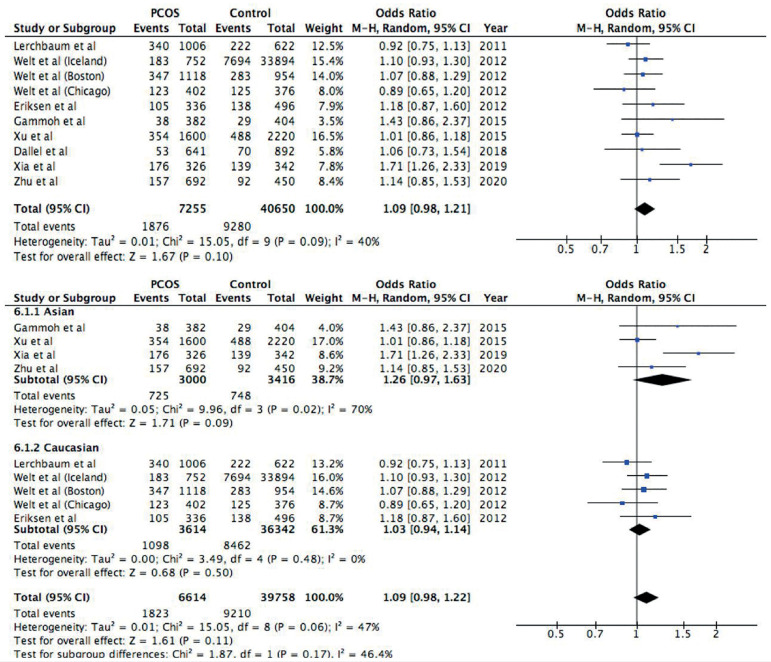
Forest plot of the association between rs2479106 polymorphism and PCOS in an allelic model (G *vs*. A). First forest plot: Total analysis of all included studies. Second forest plot: Subgroup analysis based on ethnicity, both Asians and Caucasians. Solid squares are OR from individual studies, horizontal lines are 95% CI. Pooled OR with 95% CI are presented in diamonds.

**Figure 4:**
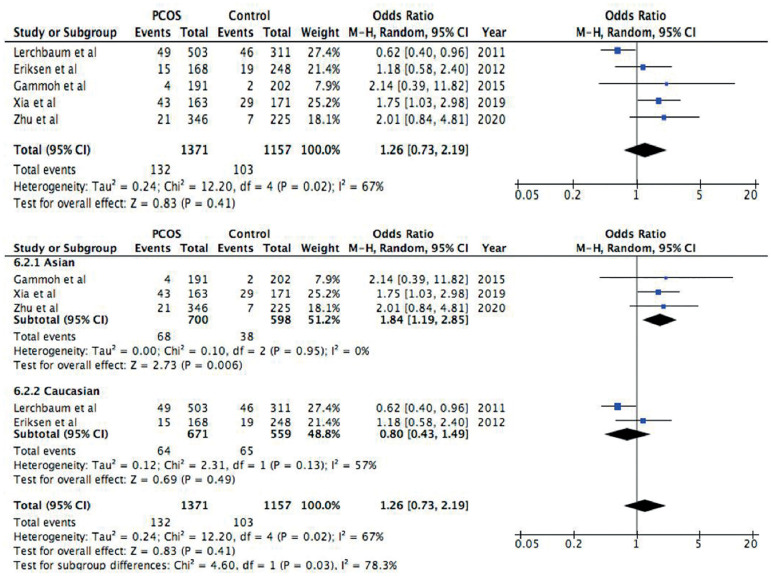
Forest plot of the association between rs2479106 polymorphism and PCOS in a recessive model (GG *vs*. GA+AA). First forest plot: Total analysis of all included studies. Second forest plot: Subgroup analysis based on ethnicity, both Asians and Caucasians. Solid squares are OR from individual studies, horizontal lines are 95% CI. Pooled OR with 95% CI are presented in diamonds.

**Figure 5:**
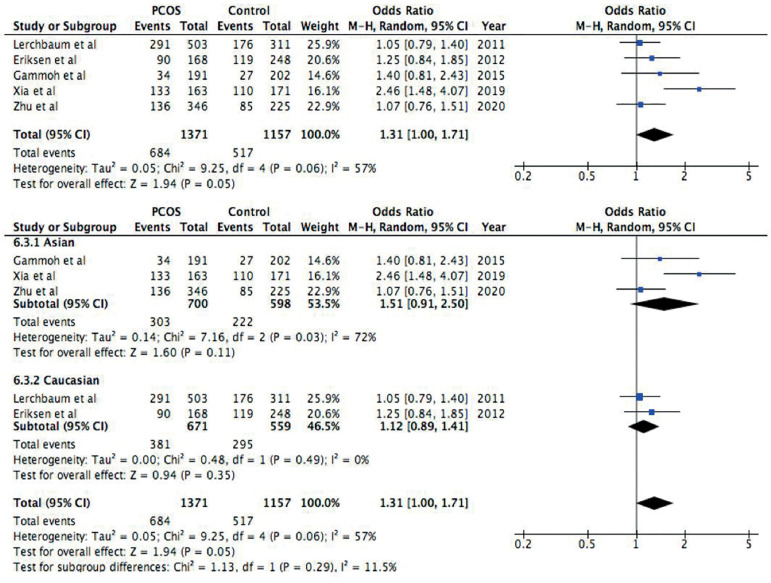
Forest plot of the association between rs2479106 polymorphism and PCOS in a dominant model (GG+GA *vs*. AA). First forest plot: Total analysis of all included studies. Second forest plot: Subgroup analysis based on ethnicity, both Asians and Caucasians. Solid squares are OR from individual studies, horizontal lines are 95% CI. Pooled OR with 95% CI are presented in diamonds.

In the study of rs2479106 polymorphism in DENND1A gene, 10 studies are included in the analysis of allele frequencies, while only five of the articles are included in the study of genotype distribution (Lerchbaum *et al.*, 2011; Eriksen *et al.*, 2012; Gammoh *et al.*, 2015; Xia *et al.*, 2019; Zhu *et al.*, 2020). Allelic-, genotype frequencies and HWE values can be found in Table 3, and the genotype distribution of the controls were all consistent with Hardy-Weinberg equilibrium. The relation between DENND1A polymorphism rs2479106 and PCOS has been analyzed with pooled OR, 95% CI, heterogeneity test and subgroup analysis, and forest plot made with Review manager are found in Figures 3, 4 and 5.

**Table 3:** Allele and genotype frequencies with HWE values of rs2479106 polymorphism in DENND1A gene.

Study	PCOS		Control		PCOS			Control			HWE
	A	G	A	G	AA	AG	GG	AA	AG	GG	
Zhu *et al*. (2020)	535	157	358	92	210	115	21	140	78	7	0.32
Xia *et al*. (2019)	150	176	203	139	30	90	43	61	81	29	0.81
Dallel *et al*. (2018)	588	53	822	70							
Gammoh *et al*. (2015)	344	38	375	29	157	30	4	175	25	2	0.31
Xu *et al*. (2015)	1246	354	1732	488							
Eriksen *et al*. (2012)	231	105	358	138	78	75	15	129	100	19	0.95
Welt *et al*. (2012) Iceland Boston Chicago	569 771 279	183 347 123	26200 671 251	7694 283 125							
Lerchbaum *et al*. (2011)	666	340	400	222	212	242	49	135	130	46	0.11

(HWE - Hardy-Weinberg equilibrium, DENND1A - Differentially Expressed in Normal and Neoplastic Development isoform 1A, PCOS - polycystic ovary syndrome).

In the allelic model (Figure 3), individuals with the minor G allele did not have a significant risk for PCOS compared to people with major A allele (OR=1.09, 95% CI=0.98-1.21, *p*=0.10). Because *p*=0.09 in the heterogeneity test, a random model was used. Furthermore, a subgroup analysis was done by ethnicity, and neither the Asian subgroup (OR=1.26, 95% CI=0.97-1.63, *p*=0.09) nor the Caucasian subgroup (OR=1.03, 95% CI=0.94-1.14, *p*=0.50) showed a significant association with PCOS. In this subgroup analysis, the study of Dallel *et al*. (2018) was not included since it was the only study with an Arab ethnicity. The heterogeneity in the Caucasian subgroup disappeared (I^2^=0%, *p*=0.48), while it remained in the Asian subgroup (I^2^=70%, *p*=0.02).

In the recessive model (Figure 4), pooled OR=1.26, 95% CI=0.73-2.19 and *p*-value=0.41, which suggest that an association with PCOS was not found in patients with this genetic model. The heterogeneity test showed heterogeneity (I^2^=67%, *p*=0.02), and a subgroup analysis was done to study the possible cause. In the subgroup analysis, an association with PCOS risk was discovered in the Asian population (OR=1.84, 95% CI=1.19-2.85, *p*=0.006), but not in the Caucasian population (OR=0.80, 95% CI=0.43-1.49, *p*=0.49).

No association with PCOS was found in the dominant model (Figure 5) of rs2479106 polymorphism (OR=1.31, 95% CI=1.00-1.71, *p*=0.05). Because of the mild heterogeneity found in this analysis (I^2^=57%, *p*=0.06), random model was used and a subgroup analysis was done. These results also indicated that this polymorphism neither has an increased risk of PCOS in Asians (OR=1.51, 95% CI=0.91-2.50, *p*=0.11) nor in Caucasians (OR=1.12, 95% CI=0.89-1.41, *p*=0.35). The heterogeneity disappeared in the Caucasian population (I^2^=0%, *p*=0.49), but remained in the Asian population (I^2^=72%, *p*=0.03).

### Sensitivity analysis

In the sensitivity analysis of the allelic model of rs6165 polymorphism in FSHR gene, the result was mildly associated with PCOS when the study of Fu *et al*. (2013) was removed (OR=1.15, 95% CI=1.01-1.30, *p*=0.03). A weak association was also seen when removing three different studies in the recessive model: Gu *et al*. (2010): OR=1.27, 95% CI=1.01-1.59, *p*=0.04; Fu *et al*. (2013): OR=1.34, 95% CI=1.04-1.71, *p*=0.02; Liaqat *et al.* (2015): OR=1.27, 95% CI=1.01-1.58, *p*=0.04. On the other hand, no individual study affected the pooled OR (95% CI) in the dominant model of this polymorphism.

Results changed when single studies were removed in every genetic model in the sensitivity analysis of rs2479106 polymorphism in DENND1A gene,. In the allelic model, a very mild association with PCOS risk appeared when Lerchbaum *et al.* (2011) was removed (OR=1.12, 95% CI=1.00-1.24, *p*=0.04). The result is statistically significant because *p*<0.05. The removal of the same study changed also the result in the recessive model (OR=1.63, 95% CI=1.12-2.36, *p*=0.01) and indicated a more obvious association with this polymorphism and the risk of PCOS. In the dominant model, Xia *et al*. (2019) changed the overall results after it was removed (OR=1.13, 95% CI=0.94-1.36, *p*=0.18), and the association with PCOS and the polymorphism disappeared.

### Publication bias

In the evaluation of funnel plots of both rs6165 and rs2479106 polymorphisms, no obvious asymmetry was found in any of the genetic models.

## DISCUSSION

FSHR gene is a familiar gene that has been studied for years. The first study that investigated the coding region of this gene back in 2001, found no mutations in Chinese Singapore patients with PCOS, and controls and patients showed similar distributions of variations in alleles (Tong *et al.*, 2001).

In this research, no association was found with polymorphism rs6165 and PCOS in any of the models. Only 1/8 studies (Kim *et al.*, 2017) showed a relation with PCOS in the allelic, recessive and dominant model. When the sensitivity analysis was done, the result was mildly changed in two models. After the removal of the study by Fu *et al*. (2013) in the allelic model, a mildly association with PCOS appeared, and a mildly association was also the result after removing three studies, one by one (Gu *et al.*, 2010; Fu *et al.*, 2013; Liaqat *et al.*, 2015).

Exactly like our result, other papers did not find a relation between PCOS and rs6165 polymorphism. In a meta-analysis from 2014, the investigation of seven studies did not find an association with PCOS in any of the models (Chen *et al.*, 2014) and the same result was also found in a research paper from 2010 (Du *et al.*, 2010). Also a third meta-analysis from 2015, where 11 studies were included, found no significant association with this polymorphism and PCOS (Qiu *et al.*, 2015). No heterogeneity was found in any of the models, so no subgroup analysis was necessary based on Asian and Caucasian ethnicity.

The polymorphism in DENND1A gene of interest in this work, rs2479106, is susceptible regarding the association with PCOS. In a study from 2012, this polymorphism was also associated with an increased risk of endometroid adenocarcinoma in patients with PCOS (Wang *et al.*, 2012). In addition, insulin levels have been investigated concerning the DENND1A gene, and other polymorphisms of DENND1A have been associated with increased insulin levels (Cui *et al.*, 2013). Several polymorphisms have been analyzed, and in a research from 2016, Gao *et al*. (2016) investigated some of them. They found relations with PCOS and polymorphisms rs10818854 and rs10986105, while the polymorphism in our study, rs2479106, only had an increased risk of PCOS in Asian patients (Gao *et al.*, 2016).

A significant association with PCOS and polymorphism rs2479106 was not found in the allelic and recessive model. In the dominant model, 95% CI=1.00-1.71 and *p*=0.05, which is insufficient to conclude if the groups are statistically significant different. Therefore, further studies have to be done to see if this model have an association with PCOS. In the allelic model, which is the model with most studies included (n=10), only one study (Xia *et al.*, 2019) showed an association with increased risk of PCOS. This article was also the only one with an association with PCOS both in the recessive (GG *vs*. GA+AA) and dominant model (GG+GA *vs*. AA).

When single studies were removed in the sensitivity analysis of this polymorphism, all the models had some changes. In the allelic model, a mild association appeared because *p*=0.04, but since 95% CI=1.00-1.24, it is insufficient to conclude because of these values. A stronger association appeared in the recessive model (95% CI=1.12-2.36, *p*=0.01) when removing the study of Lerchbaum *et al*. (2011), while the association from the dominant study, disappeared completely when removing the study of Xia *et al*. (2019) (95% CI=0.94-1.36, *p*=0.18). Because the models were sensitive to the removal of only one study, additional research have been done to strengthen the result.

Other meta-analysis have also been investigating this polymorphism. A study from 2016 indicated that patients with rs2479106 polymorphism have increased risk to get PCOS compared to controls in allele-, heterozygote- and dominant genetic model (Bao *et al.*, 2016). Differently, another paper from 2016 did not find a significant association between patients with PCOS and controls in the allelic model of rs2479106 (Gao *et al.*, 2016).

All three models did show moderate heterogeneity (allelic model: I^2^=40%, *p*=0.09; recessive model: I^2^=67%, *p*=0.02; dominant model: I^2^=57%, *p*=0.06), and subgroup analysis was done in each of them. In the allelic model, four articles were included in the Asian population, while five of them had Caucasian background. No association was found with PCOS in any of them. The heterogeneity did disappear in the Caucasian one, while the Asian population went from moderate to high heterogeneity (I^2^=70%, *p*=0.02), which must be due to other reasons than ethnicity.

In the subgroup analysis in the recessive model, the Asian population showed an association with increased risk of PCOS (95% CI=1.19-2.85). The heterogeneity disappeared in the Asian subgroup (I^2^=0%), while it remained a moderate heterogeneity in the Caucasian subgroup (I^2^=57%). The Asian population consisted of three studies, while only two populations were Caucasian, which is an extremely low number to have in a subgroup analysis. In addition, the value of the overall heterogeneity in the subgroups (I^2^=78.3%), indicate that the subgroups are different from one another, and that there are still differences in the same ethnicity. Low number of studies were also the case in the dominant subgroup analysis based on ethnicity, and the heterogeneity remained in the Asian population.

This meta-analysis has also its limitations. First, heterogeneity was found in all models in the study of DENND1A rs2479106 polymorphism. In the analysis of Caucasian population, heterogeneity remained in the recessive model, which also was the case with the Asian population in the analysis of the allelic model. This can for example be due to other factors not counted for in this meta-analysis, like obesity, BMI or errors with methods and genotyping in each study. Second, a small number of studies are included, especially in subgroup analysis of the recessive and dominant model in DENND1A gene rs2479106 SNP (three studies with Asian origin and two studies with Caucasian origin). A low number of studies can weaken the statistical power of the analysis and can increase the false negative or false positive results, and it need to be interpreted with caution. Third, the ethnicities of the included articles were only Asian and Caucasian. No studies from Africa were found, and a broader investigation based on ethnicity should be done in the future to see if genetic origin matter. Fourth, only English articles were included, and relevant articles and results can therefore be missed. A search in multiple languages, especially in Chinese, could have improved the outcome due to their broad research in this field.

## CONCLUSION

To conclude, DENND1A rs2479106 polymorphism was the only gene in this study with a relation to PCOS, but it was only seen in the recessive model in patients with Asian ethnicity. It is unclear to say, since only one model was affected, if this gene is a part of the pathogenesis of PCOS or not. On the other hand, no associations were found between PCOS and rs6165 polymorphism in FSHR gene. Limitations like small samples sizes, heterogeneity within ethnicities and variable results from sensitivity analysis can be found within the polymorphisms, and these results must be interpreted with caution. Absence of ethnicities other than Asian and Caucasian will narrow down the diversity of patients and can increase the false negative or false positive results in a global perspective. In future researching, less heterogeneity and bigger sample sizes with a broader ethnic origin should be included to strengthen the statistical power and to get a better understanding of this common heterogenous syndrome found in reproductive women.

## Abbreviations:

PCOS: polycystic ovary syndrome
FSHR: follicle stimulating hormone receptor
DENND1A: differentially expressed in normal and neoplastic development isoform 1A
OR: odds ratio
CI: confidence interval
SNP: single nuclear polymorphisms
Thr: threonine
Ala: alanine
